# Effects of surgical repair of obstetric fistula on severity of depression and anxiety in Ethiopia

**DOI:** 10.1186/s12888-019-2045-3

**Published:** 2019-02-07

**Authors:** Bekele Belayihun, Azwihangwisi Helen Mavhandu-Mudzusi

**Affiliations:** 1 Department of Health Studies, University of South Africa, Addis Ababa, Ethiopia; 2 Department of Health Studies, University of South Africa, Pretoria, South Africa

**Keywords:** Anxiety, Depression, Ethiopia, Obstetric fistula, Surgical repair

## Abstract

**Background:**

The surgical repair of fistula can address the physical symptoms, but may not end the psychological challenges that women with fistula face. There are a few studies that focus on women with this condition in Ethiopia. Hence, the aim of this study was to determine the effects of surgical repair of obstetric fistula on the severity of depression and anxiety in women with obstetric fistula in Ethiopia.

**Method:**

The study employed a longitudinal study design to investigate the changes in 219 women with obstetric fistula admitted to six fistula management hospitals in Ethiopia. The data were collected on admission of the patients for obstetric fistula surgical repair and at the end of six-month post repair. A structured questionnaire was used to obtain socio-demographic information and medical history of the respondents. Depression and anxiety symptoms were measured using the Patient Health Questionnaire (PHQ-9) and General Anxiety Disorder (GAD-7) scales. The data was entered using Epi-Data software and then exported to SPSS for further analysis. The Mann-Whitney-U test, the Kruskal-Wallis test and Paired t-test were performed to measure the change in psychological symptoms after surgical repair.

**Result:**

Though 219 respondents were interviewed pre-obstetric fistula surgical repair, only 200 completed their follow up. On admission, the prevalence of depression and anxiety symptoms were 91 and 79% respectively. After surgical repair, the prevalence rate was 27 and 26%. The differences in the prevalence of screen-positive women were statistically significant (*P* < 0.001).

**Conclusion:**

The study concluded that the severity of depression and anxiety symptoms decrease post-obstetric fistula surgical repair. However, a woman with continued leaking after surgery seems to have higher psychological distress than those who are fully cured. Clinicians should manage women with obstetric fistula through targeted and integrated mental health interventions to address their mental health needs.

## Background

An obstetric fistula is an abnormal opening between a woman’s vagina and bladder and/or rectum through which her urine and/or faeces continually leak [1]. It is caused by continuous pressure exerted on the vaginal wall, bladder, and/or rectum, by the impacted head of the flutes, which leads to decreased circulation and breakdown of vaginal tissue. Over time, the tissue gives way, leaving a hole through which urine or stool leak uncontrollably. Several factors contribute to increasing obstetric fistula, include; a delay in seeking delivery service, prolonging labour, cultural practices, stillbirth, poor access to maternal health care, malnutrition, unsafe abortion, and sexual violence [2–6]. Previous studies recognize that psychological distress is influenced by traumatic events such as; cause of obstetric fistula, level of social support, personal factors such as socio-demographic status, childhood development factor, and economic status [7, 8].

Women with obstetric fistula are socially stigmatised and marginalised, psychologically affected and economically deprived, and often suffer a traumatic birth experience [9–11]. These situations are correlated with mental health problems [12–14]. Obstetric fistula associated with mental health problems is one of the most burdensome diseases among women in their early productive years. Studies in low-income countries, including Ethiopia, report that women with fistula have a significantly higher incidence of symptoms of depression, psychosocial dysfunction and anxiety [11, 15].

Obstetric fistula repair surgery has a positive effect on women’s lives through improvements in their physical and psychological condition, as well as their interpersonal relationships [16]. There is preliminary evidence suggesting that fistula repair surgery leads to a decrease in psychological symptoms. These women display experiences of improved quality of life and social functioning, and decreased psychological distress from admission to post-surgery [17–19]. In spite of the significant positive effect of surgery on psychological distress, there is also evidence that some women with obstetric fistula continue to experience distress after repair [16, 19, 20]. While becoming content after repair represents a rebirth for women suffering from fistula, failure of fistula closure can lead to further depression and isolation [21].

In the aforementioned evidence, there was a significant difference in post-surgery psychological distress relief and social functioning among women whose repairs fail compared to those whose repairs are successful [19]. Unsurprisingly, women whose surgeries resulted in incomplete fistula closure or stress incontinence reported persistent negative moods following fistula repair [22]. Most of the time, health care professionals usually focus on physical treatment whilst ignoring the associated mental health problems such as depression and anxiety. The continued focus on physical intervention/treatment means that women will continue to suffer the salient burden of the associated mental health ailments, which can compromise the recovery process. Despite this burden, which is prevalent in most of the developing sub-Saharan Africa countries including Ethiopia [11, 15], there is a limited amount of literature on the effects of the surgical repair of obstetric fistula on the severity of depression and anxiety. Thus, this study determines the effects of surgical repair of obstetric fistula on the severity of depression and anxiety in Ethiopia.

## Methods

### Design

The study employed longitudinal design to detect changes in the severity of depression and anxiety and investigate the relationship between mental health and surgical repair outcomes of obstetric fistula.

### Study setting

The study was conducted in six fistula management hospitals in Ethiopia, located in Amhara, Tigray, Oromia, Addis Ababa, Harar and SNNPR. These hospitals are a world-class centre of excellence for treating women with obstetric fistula. These six hospitals were built exclusively for managing fistula cases. They provide surgical repair of obstetric fistula free of charge through the support of governmental legislation and a non-governmental organisation (NGO).

### Sample size determination

To determine the appropriate sample size, different measures were considered, such as controlling the Type I error (α = 0.05), Type II error (β = 0.2), correlation (effect size) = 0.3, and the ratio of indicators to a latent variable while controlling the family-wise error rate = 3 [23]. The calculated minimum sample size was 200. However, as previous literature shows that large sample sizes are critical in measuring the effect, there were reservations about the sample size above lacking adequate representation to detect changes. Because of this, all newly registered women with a physician-confirmed diagnosis of fistula and scheduled surgical repair admitted to the six fistula hospitals within a six-month period (from 1 January to 31 June 2017), were included.

### Sampling technique

All eligible individuals were recruited to participate in the study consecutively until the required sample size was reached. Consecutive sampling is a type of non-random sampling, where each respondent that meets the eligibility criteria is recruited to participate [24, 25]. To be eligible, the participants had to be women who had a minimum of three months’ experience with fistula; newly registered with a physician-confirmed diagnosis of fistula and who were awaiting obstetric surgical repair. A total of 320 individuals visited the six fistula hospitals for obstetric fistula care during a six month period. One hundred and one (101) of them were excluded because they did not meet one or more of the eligibility criteria; i.e. they had fistula for less than three months (*n* = 24); their cases were not new (being retreated or undergoing repeated surgery) (*n* = 49); or their condition was not an obstetric fistula (*n* = 28). Ultimately, 219 individuals fulfilled the eligibility criteria to participate in the study. All eligible individuals (*n* = 219) were willing to participate and provided their consent during their admission, but only 200 eligible individuals completed the follow-up process. Nineteen respondents chose to exercise their right to withdraw from the study at any time and are therefore, were not included in the analysis. The dropout rates were acceptable compared to clinical studies in general [26, 27]. The study participants, women diagnosed with fistula, were recruited from multiple hospitals and participated in the study [28].

### Recruitment

The interviews were conducted at the fistula hospitals using structured and pre-coded questionnaires. The researchers selected six-midwives and six psychiatric nurse research assistants. The researchers provided three days of training to research assistants on how to approach respondents, obtaining consent, administration of questionnaires, and research ethical principles. The questionnaire was administered two times (first time during the admission and a second time during discharge). Midwife nurses running obstetric fistula outpatient departments screened people for eligibility, explained the aim of the research project, and invited them to participate in the study. When the person expressed interest to participate, the research assistants interviewed their them after getting their consent. Respondents were contacted within two days of admission to the fistula ward and informed that they will be contacted for a follow-up interview after getting treatment.

### Data collection tool

To collect relevant data, interviews were conducted at the fistula hospitals using a structured and pre-coded questionnaire. The questionnaire was structured into three parts: (1) socio-demography; (2) obstetric and gynaecologic history; and (3) symptoms of the psychological disorder (depression and anxiety). The questionnaire covered various variables and measurements. The researchers measured severity of depression and anxiety symptoms using the Patient Health Questionnaire nine-item (PHQ-9) and Generalized Anxiety Disorder Seven-item (GAD-7) assessment, respectively. The measurement item (PHQ-9 and GAD-7) is widely used in different research and validated in different settings and population groups [28–31]. PHQ-9 and GAD-7 has been validated in Ethiopia using different cut-off points (cut-off point five or above and cut-off point 10 or above) [29, 32–34]. We chose the score cut-off point of five or above to define the mean severity of depression and anxiety symptoms. In this study, we used the terms depression and anxiety to mean severity of depression and anxiety for the simplicity purpose. The questionnaire was translated into local languages (Amharic, Afaan Oromo & Tigrigna), and was translated back into English to check its consistency.

### Data collection

The research assistants collected baseline data using a structured questionnaire before the women underwent obstetric fistula surgery. The initial interviews were conducted under researchers’ guidance and supervision. All the women were subsequently treated surgically and had a two-week post-operative recovery period with free bladder drainage through an indwelling Foley catheter. At the end of the two weeks, the catheter was removed and the clinical outcome was recorded. Upon discharge, the depression and anxiety symptoms measurement scale was administered again, and fistula cure perceptions (opinions) of respondents were included, but this time, the questionnaire was administered by psychiatric nurse research assistants who were blind to the responses of the first round of questionnaire in order to eliminate the interviewer effect or bias, and in order to free the respondents from possible fear of judgment. The researchers’ made regular visits (once every week) to the hospitals to monitor data collection. Supervision meetings were also held with data collectors every month.

### Data management and analysis

The researchers assessed the quality, accuracy and completeness of the collected data using range plausibility and cross-validation checks. Data were checked, coded and entered into Epi-Data version 3.2. The accuracy of data entry was checked by running frequency analysis and making range checks every time data were entered. The data entry errors were corrected by cross-checking with the completed questionnaires. The principal investigators using Epi-Data calculated the dates for the second round assessments of each respondent. After completing the data entry, the data were exported to SPSS version 20 for analysis. Investigators tried to determine whether the somatic symptoms of depression and anxiety were measuring the same underlying constructs as the cognitive and emotional symptoms. In preparation for factor analysis, investigators checked the PHQ-9 and GAD-7 data for the presence of adequate corrections among the items and adequacy of the sample. Explanatory factor analysis was carried out with maximum likelihood extraction and oblimin rotation, and the numbers of factors were determined using eigenvalue, scree plot and parallel analysis. Internal consistency was checked using Cronbach’s alpha. The prevalence and severity of depression and anxiety symptom among women with obstetric fistula at baseline were determined by computing the proportion of respondents scoring five or more on the PHQ-9 and GAD-7 scale. The result of the PHQ-9 and GAD-7 were analysed using the Mann-Whitney_U test, Kruskal Wallis test, and Paired t-test.

## Results

### Socio-demographic characteristics

The study included 219 eligible women with obstetric fistula. The mean age of the respondents was 28 years, ±7.7 standard deviations (SDs). Approximately 36% of the respondents were in the 26–30 year age bracket, while 17% of the respondents were under 21 years. The majority of the respondents (85%) were rural residents. Of all the respondents, Orthodox Christians constituted 34%, followed by Muslims at 32% and Protestants at 31%. Most of the respondents (74%) had not attended any level of education (unable to read and write). Almost all of the respondents were at some point married (93%), of which 20% were divorced/widowed at the time of the study, while 13% were not living with their sexual partners/husbands (Table 1).

**Table 1:** Socio-demographic characteristics of women with obstetric fistula in Ethiopia (*n* = 219)

Characteristic	Number	Percent (%)
Current Age		
< 25+	77	35.2
26–30	79	36.1
> 31+	63	28.8
Residence		
Urban	31	14.1
Rural	188	85.8
Educational status		
Unable to read and write	162	74.0
Read and write or Primary	57	26.0
Religion		
Orthodox	75	34.2
Muslim	69	31.5
Protestant	68	31.1
Others	7	3.2
Ever married		
Yes	203	92.7
No	16	7.3
Current marital status		
Married	159	72.6
Divorced/Widowed	44	20.1
Single	16	7.3

### Gynaecologic and obstetric history of women with obstetric fistula

At the time of the study, most respondents (60%) were living with their sexual partners/husbands, and the mean numbers of children were 2.8±, 2.6SD. The mean ages of respondents at their first marriage and first delivery were 16 years ±3.1 SD and 19 years ±3.6 SD, respectively. A total of 13% of the respondents had their first child when they were less than15 years old, and 57% of them had their first child when they were 16–20 years old (Table 2). Only 38.8% of respondents had lived with obstetric fistula for more than one year, whereas the rest (61.2%) had lived with obstetric fistula for a year or less. Most of the respondents (72.6%) reported that they had a labour duration of 24 or more hours. Thirty-seven percent (37%) of the respondents had delivered through a Caesarean section (Table 2).

**Table 2:** Gynaecologic and obstetric history of women with obstetric fistula in Ethiopia (*n* = 219)

Characteristic	Number	Percent (%)
Age at 1st marriage (year)		
> 18	108	49.3
18+	111	50.7
	Mean ± SD	19.25 ± 3.6
Age at first delivery (Year)		
> 18	67	30.6
18+	152	69.4
	Mean ± SD	19.25 ± 3.6
Disease duration (year)		
< 1+	134	61.2
1–5	46	21.0
> 5	39	17.8
	Mean ± SD	2.3 ± 3.5
	Median	0.75
Have you ever given birth?		
Yes	215	98.2
No	4	1.8
Antenatal attendance (last child)		
0	87	40.5
1–3	63	29.3
4+	65	30.2
Number of live births		
0	39	18.1
1–2	83	38.6
3–5	61	28.4
> 5	32	14.9
Labour duration (last delivery in hours)		
< 24	59	27.4
24+	156	72.6
Mode of delivery (last delivery)		
Normal vaginal delivery	94	43.7
CS delivery	81	37.7
Other mode of delivery	40	18.6
Place of delivery (last delivery)		
Home	63	29.3
Hospital	127	59.1
Health Center	20	9.3
Health post	5	2.3
Delivery outcome (last birth)		
Stillbirth	141	65.8
Live birth	74	34.4
Number of Stillbirth experiences		
0	62	28.8
1	114	53.0
2+	39	18.1

### PHQ-9 and GAD-7 scale reliability test

The PHQ-9 and GAD-7 had a clear single-factor structure explaining 34.9 and 26% of the variance respectively, based on the explanatory analysis. The loading items of depression ranged from 0.49 to 0.72 and anxiety ranged from 0.57 to 0.72. Overall, the PHQ-9 items showed good internal consistency (Cronbach’s alpha =0.808) and test re-test reliability (intraclass correlation coefficient = 0.813). The anxiety measurement scale (GAD-7 items) had also a good internal consistence (Cronbach’s alpha = 0.82) and test re-test reliability (intraclass correlation coefficient = 0.73). The PHQ-9 and GAD-7 items appear to be a reliable and valid instrument used to measure depression and anxiety disorders among women with obstetric fistula in Ethiopia. The sample size of the study was appropriate to proceed with further analysis (Bartlett’s test of sphericity (*p* < 0.001) & KMO = 0.881).

### The prevalence and severity of depression and anxiety among women with obstetric fistula awaiting surgical repair

Women with obstetric fistula usually experience a significant number of potentially traumatic cases and exhibit significant symptoms of depression and anxiety. The prevalence of depression symptom in women with obstetric fistula using a cutoff value of five or above was 91% (95% CI = 85.4, 94%), of which 33.3% scored in the mild depression range, whereas 11% were classified as experiencing severe depression. The prevalence of anxiety symptom in women with obstetric fistula using a cutoff value of five or above was 79% (95% CI = 75,83%), of which 32% had mild anxiety and 20% had severe anxiety, even after controlling of the underlying risk factors for psychopathology (Table 3).

**Table 3:** Prevalence and severity of depression and anxiety among women with obstetric fistula awaiting surgical repair in Ethiopia (*n* = 219)

Psychosocial variable	Depression	Anxiety
	*Number (%)*	*Number (%)*
Over all prevalence	200 (91.3)	174 (79.5
Level of severity		
No symptom/Minimal	19(8.7)	45 (20.5)
Mild	73 (33.3)	71 (32.4)
Moderate	53 (24.2)	59 (26.9)
Moderately severe	49 (22.4)	
Severe	25 (11.4)	44 (20.1)

### Changes and variation in the severity of depression and anxiety between pre and post-surgical phase

Initially, 219 eligible respondents were recruited upon their admission, but only 200 respondents completed the follow-up process and responded to the questionnaire upon discharge, following the surgical repair of their obstetric fistula. Upon discharge, the prevalence of depression symptoms was 27%, of which 1% was severe and were 16% mild. A total of 73% of women had no or minimal depression symptoms. The prevalence of anxiety symptoms was 26%. Regarding the severity of anxiety, 16% of women had mild anxiety, 75% had no or minimal anxiety symptoms and 6% had severe anxiety symptoms. The difference in prevalence of screen-positive women was statistically significant (paired test, *p* < 0.001) as was the difference in mean score (Mann–Whitney U test, *p* = 0.000) (Table 4). In exploratory analysis, the study detected the association between severity of leaking and severity of depression and anxiety symptoms at follow-up. Of our sample, 69% of the respondents believed that their fistula was cured during follow-up interviews. Ultimately, the findings showed that, the severity of leaking was positively associated with psychological distress (depression and anxiety) (*p* < 0.05), (Table 5).

**Table 4:** Psychological symptoms (depression and anxiety) between pre and post-surgical follow-up among women with obstetric fistula in Ethiopia (*n* = 200)

Symptoms	Baseline *M (SD)*	Follow-up *M (SD)*	Paired test (2 tailed)		Kruskal Wallis test		Mann-Whitney Test	
			*T*	*P-value*	X^2^	*P-value*	z	*P-value*
Depression	12.29 (5.9)	3.64 (4.6)	15.94	0.000^*^	178.1	0.000	−13.3	0.000^*^
Anxiety	9.90 (5.2)	3.35 (4.7)	13.29	0.000*	145.5	0.000	−12.1	0.000^*^

*P < 0.001

**Table 5:** Exploratory association between cure with psychological symptoms after post-surgical repair among women who completed surigical repair treatment in Ethiopia (*n* = 200)

Variable	Cure status after surgical repair		Group difference (t-test)	*P*-value
	Cured *M (SD)*	Not Cured *M (SD)*		
Depression symptoms	1.16 (2.5)	3.9 (4.3)	−4.3	0.000**
Anxiety symptoms	1.24 (2.6)	3.4 (4.3)	−3.3	0.002*

*p < 0.01, ** *p* < 0.001

## Discussion

There is a little existing preliminary evidence suggesting that women with obstetric fistula experience a decrease in psychological distress following fistula repair surgery. In this study, the difference in anxiety and depression symptoms before and after the surgical repair was statistically significant. Follow-up interviews after surgical repair revealed that over time, (from admission to 5.64 weeks) women who had fistula conditions repaired reported a decrease in depression and anxiety symptoms. Results from the follow-up study verify previous literature that has suggested an overall improvement in the mental health outcome and quality of life of patients following obstetric fistula repair [17, 19, 30]. Qualitative findings corroborate this evidence, showing that women with obstetric fistula often recount experiences of improved quality of life and overall level of happiness after fistula repair [18]. The current study added to this literature by specifying and quantifying the depression and anxiety symptoms that change following surgical repair. The sharp decline in these symptoms suggests that women with obstetric fistula generally experience improved mental health following surgical repair. The current and previous findings suggest that overall, women with obstetric fistula experience improvement in mental health following fistula repair. The decrease in anxiety and depression symptoms at follow-up was rather remarkable, given the level at which symptoms were reported at baseline. This is not a typical course for anxiety and depression unless traumatic stress is conceptualized as chronic and ongoing for women with obstetric fistula rather than restricted to a single event (the traumatic childbirth).

The post-operative score was highly dependent on the success of the surgical repair. Although some women do not recover fully on the first surgical repair of their fistula and will continue to experience symptoms or may need additional surgery, many women with obstetric fistula do benefit from surgical repair [16]. Following surgery, 18 of the 200 repairs were considered to have failed. Of the remaining, 69% (137) of the respondents reported that their fistula was cured and 22.5% (45) of them reported that it was not cured. Of those who reported that their fistula was not cured, 5% (10) leaked only with extraction, 13% (26) leaked only while walking, but were dry while sitting, 4.5% (9) leaked while walking and occasionally while sitting. The study showed a significant difference in depression and anxiety symptoms among respondents who did and did not continue to experience leaking after completing their follow-up treatment. Respondents who did not perceive themselves as cured had significantly higher depression and anxiety symptoms than those who believed they were cured (Table 5). Similar evidence argued that the failure of fistula closure can lead to further depression, anxiety, and isolation [21]. In fact, continued leaking could potentially be considered an ongoing traumatic event. If obstetric fistula and the consequences thereof entail ongoing trauma, then successful surgery should put an end to it. Thus, a decrease in anxiety and depression symptoms following repair surgery may indicate a natural healing process in the wake of trauma.

There were several limitations that should be considered when reviewing these results. The first limitation is that the study measures the change of psychological distress only from admission to immediately prior discharge after surgical repair. This excluded the long-term mental health outcomes of women with obstetric fistula, which might occur after discharge. Another limitation of the study is that while the time of pre and post measuring of depression and anxiety was short, the follow-up assessment was also limited unequal duration of stay at the hospital between the date of admission and the date of discharge, and this might affect the exact change of patient’s mental health outcome. Additional limitations are related to a comparison group of causal relationship (cured vs not cured). The study measured the casual relationship between severity of leaking and severity of psychological distress (depression and anxiety) with considering internal comparison groups (cured versus those who are not cured) by interviewing the respondents, without considering a clinical diagnosis or assessment of associated functional impairment. The symptoms might be high or low than the actual symptoms. Politeness bias, especially as it relates to measures experience of leaking or failure of fistula closure after completing their follow up may affect these results. The respondents may not know exactly the causes of their incontinence (leaking), whether they leaked due to incomplete fistula closure or due to stress incontinence. This type of bias may result in higher or lower levels of reported than actually repaired. This bias may reduce the validity of the findings. Finally, women with obstetric fistula may have been more or less likely to provide honest responses to some of the sensitive topics such as; “feeling afraid”, “Being restless”, “Feeling bad about yourself” and “Thoughts that you would be better off dead” because of face-to-face interviews. The investigators tried to increase honest responses by hiring female interviewers who also had previous experience working with women with obstetric fistula. All interviews were conducted with privacy to assure respondents that their responses were private and confidential.

## Conclusion

The measures of the severity of depression and anxiety symptoms showed improvement in post-obstetric fistula surgical repair when compared to the baseline findings. The follow-up data showed that women with obstetric fistula reported a decrease in the severity of depression and anxiety symptoms from their admission to their discharge after surgical repair. The severity of leaking was positively associated with depression and anxiety. After surgery, the severity of depression and anxiety symptoms appear to decrease in general. However, women with continued leaking experienced more psychological distress than those who were fully cured. Fistula clinicians should seek strategies to address psychological problems in their patients through targeted integrated mental health interventions to address their mental health needs. The study showed that some of the respondents continued leaking (incontinence) after surgical repair and increased psychological symptoms, warrants further investigation using clinical evaluation and most appropriate control groups might be a warrants to determining the association between psychological symptoms and incontinence.

## Abbreviations

CI: Confidence Interval
GAD-7: General Anxiety Disorder-seven items
NGO: Non-Governmental Organization
PHQ-9: Patient Health Question-nine items
SD: Standard Deviation
SNNPR: South Nation Nationality People Regions
